# O5-2 Tele-rehabilitation program ‘Funktionstraining-online' in people with Multiple Sclerosis - preliminary results of a first online survey

**DOI:** 10.1093/eurpub/ckac094.034

**Published:** 2022-08-29

**Authors:** Anne-Marie Gemmerich, Woschek Stephanie, Patrick Schubert, Nadine Scholl, Charlotte Klump, Haas Christian

**Affiliations:** 1 Institute of complex research, Hochschule Fresenius, University of Applied Science, Idstein, Germany; 2 Regional association Hessen, German Multiple Sclerosis Society, Frankfurt am Main, Germany

**Keywords:** tele-rehabilitation, physical exercise, multiple sclerosis, survey

## Abstract

**Background:**

In 2019, the German Multiple Sclerosis (MS) Society was certified as a provider for ?Funktionstraining? (FT). FT is an exercise training program developed for people with disabilities or people at risk of disability, takes place weekly and is led by a qualified FT-therapist. It is prescribed by a physician (between 12 and 24 months) and is part of outpatient medical rehabilitation. Due to the COVID-19 pandemic, the tele-rehabilitation concept ‘Funktionstraining-online’ (FT-online) was developed for pwMS. This study examines the FT-online from a participants' point of view within an online survey.

**Methods:**

We developed an online questionnaire that included data of sample characteristics, participants' experiences with FT-online in terms of usability and training content. Further, the survey captured the quality of FT-online and the FT-therapist and compared carefully FT-online with face-to-face training. The standardised questionnaires Self-Efficacy Scale - Short Form (ASKU), the Multiple Sclerosis Questionnaire for Physiotherapists MSQPT and the Fatigue-Questionnaire (WeiMuS) were included. The 103 interviewed people with MS (pwMS) participated in FT-online at least for three months.

**Results:**

Data analyses are in process. The response rate was 75% (n = 78). The analysed sample is 53.4±8.1 years old with a disease duration of 18±8.7 years (male=7, female=71). Preliminary results in the ASKU certify a low self-efficacy in the sample (2.25 ±0.67). Qualitative results show the advantages for FT-online in the independence of mobility and infrastructure, flexibility, compatibility within job and family, group feeling in times of social distancing and an increase in quality of life. The disadvantages described by the sample are mainly technical problems and lack of socialising. 35% (n = 27) participants experienced both: FT-online and face-to-face. 44.4% (n = 12) of this sample prefer FT-online, 33.3% (n = 9) prefer the face-to-face training course and 22.2% (n = 6) rate both kinds of training equal.

**Conclusions:**

The implementation of FT-online was an important step to ensure the exercise-related physical activity in pwMS during the COVID-19 pandemic. The descriptive analyses show high potential for FT-online training in pwMS. We are aware of the bias in current data, because of the pandemic situation both the free choice of FT-offers and the pandemic circumstances held influence in study outcomes. First results lead to the conclusion that FT-online may be beneficial for a broad range of persons with neurologic diseases, movement disorders and fatigue episodes and should be considered for future medical rehabilitation.

